# Supplementary material for: Tempo: an unsupervised Bayesian algorithm for circadian phase inference in single-cell transcriptomics
Source: Nat Commun. 2022 Nov 2;13:6580. doi: 10.1038/s41467-022-34185-w (PMC9630322; doi:10.1038/s41467-022-34185-w)
Supplement: Supplementary file 3 — Description of additional Supplementary File [file 41467_2022_34185_MOESM3_ESM.pdf]

<u>Descriptions of additional Supplementary files</u>

Supplementary Dataset 1: SMC dataset
Zipped .h5ad object for the aortic smooth muscle cells which can be loaded using the AnnData or ScanPy libraries in Python. The object contains cell information in the .obs slot. In particular, cell collection times and cell library sizes can be viewed in the 'zt' and 'lib_size' columns of the .obs slot. Gene information can be found in the .var slot. The object contains UMI counts in the .X slot ([cells x genes] matrix of the raw counts).

Supplementary Dataset 2: Fibroblast dataset
Zipped .h5ad object for the aortic fibroblasts which can be loaded using the AnnData or ScanPy libraries in Python. The object contains cell information in the .obs slot. In particular, cell collection times and cell library sizes can be viewed in the 'zt' and 'lib_size' columns of the .obs slot. Gene information can be found in the .var slot. The object contains UMI counts in the .X slot ([cells x genes] matrix of the raw counts).

Supplementary Dataset 3: Endothelial dataset
Zipped .h5ad object for the aortic endothelial cells which can be loaded using the AnnData or ScanPy libraries in Python. The object contains cell information in the .obs slot. In particular, cell collection times and cell library sizes can be viewed in the 'zt' and 'lib_size' columns of the .obs slot. Gene information can be found in the .var slot. The object contains UMI counts in the .X slot ([cells x genes] matrix of the raw counts).

Supplementary Dataset 4: Macrophage dataset
Zipped .h5ad object for the aortic macrophages which can be loaded using the AnnData or ScanPy libraries in Python. The object contains cell information in the .obs slot. In particular, cell collection times and cell library sizes can be viewed in the 'zt' and 'lib_size' columns of the .obs slot. Gene information can be found in the .var slot. The object contains UMI counts in the .X slot ([cells x genes] matrix of the raw counts).
